# Serum Thyrotropin Is Positively Correlated with the Metabolic Syndrome Components of Obesity and Dyslipidemia in Chinese Adolescents

**DOI:** 10.1155/2014/289503

**Published:** 2014-08-21

**Authors:** Jingfan Zhang, Ranhua Jiang, Ling Li, Ping Li, Xue Li, Zinan Wang, Liang Li, Weiping Teng

**Affiliations:** ^1^Department of Endocrinology, Shengjing Hospital of China Medical University, Shenyang, Liaoning 110004, China; ^2^Liaoyang Diabetes Hospital, Liaoyang, Liaoning 111000, China; ^3^Liaoning Province Key Laboratory of Endocrine Diseases, Shenyang 110001, China; ^4^Department of Endocrinology, The First Hospital of China Medical University, Shenyang, Liaoning 110001, China

## Abstract

Metabolic syndrome is a medical disorder characterized by obesity, hyperglycemia, dyslipidemia, and hypertension. Thyroid hormone has been shown to affect many metabolic processes. This study was undertaken to explore the relationship between serum thyrotropin and components of metabolic syndrome in Chinese adolescents. Waist circumference (76.4 ± 10.7 versus 70.0 ± 10.6 cm, *P* = 0.006) and body mass index (23.90 ± 4.20 versus 21.51 ± 4.16 kg/m^2^, *P* = 0.011) were significantly greater among adolescents with subclinical hypothyroidism compared with euthyroid subjects. The risk of obesity in the subclinical hypothyroid group was 3.444 times that in the euthyroid group (odds ratio = 3.444, 95% confidence interval (CI): 1.570–7.553). Serum TSH was significantly positively correlated with waist circumference (*β* = 1.512, *P* = 0.019), TC (*β* = 0.160, *P* = 0.003), LDL-C (*β* = 0.032, *P* = 0.008), and TG (*β* = 0.095, *P* = 0.001). The TSH level in the metabolic syndrome group was significantly higher than that in nonmetabolic syndrome group (2.65 [2.28–3.80] versus 2.53 [1.92–3.45] mIU/L, *P* = 0.032). Serum TSH within the reference range was positively associated with TC (*β* = 0.173, *P* = 0.013), LDL-C (*β* = 0.031, *P* = 0.043), and TG (*β* = 0.132, *P* = 0.021). Increased serum TSH in adolescents may be a potential risk factor for metabolic syndrome.

## 1. Introduction

The prevalence of metabolic syndrome and obesity has continued to increase worldwide in recent years. According to the guidelines of the US National Cholesterol Education Program (NCEP), the age-standardized prevalence of metabolic syndrome is 9.8% in men and 17.8% in women in China [1]. Using a similar definition, Wan et al. [2] screened overweight and obese children from a group of nearly 2000 children, aged 6–18 years in Beijing, China, and found the prevalence of metabolic syndrome to be 29.8% among obese children.

Thyroid hormones have been shown to affect many metabolic processes, and overt hypothyroidism is clearly related to body weight and greater adiposity [3]. Even subclinical hypothyroidism, which is considered as a mild thyroid disorder, has been associated with both hypertension [4] and dyslipidemia [5]. Metabolic syndrome is a medical disorder characterized by a cluster of obesity, hyperglycemia/insulin resistance, dyslipidemia, and hypertension. To date, several studies have demonstrated a correlation between thyroid function and components of metabolic syndrome in adults [6–8]. However, whether such a relationship exists in adolescents remains unknown. In this present study, 919 students aged 11 to 16 years old were enrolled to determine whether a correlation exists between thyrotropin (TSH) levels and the components of metabolic syndrome in adolescents.

## 2. Materials and Methods

### 2.1. Study Population

A total of 936 students (aged 11–16 years; 46.6% female) from junior and senior high schools in Liaoyang, a medium-sized city in northeastern China, were initially recruited for the study after informed consent was obtained from their parents or legal guardians. The study and research protocols were approved by the Medical Ethics Committee of Shengjing Hospital of China Medical University.

Participants with a history of thyroid disease under thyroxine or antithyroid drugs treatment, those taking medications affecting thyroid function, those with overt hyperthyroidism or overt hypothyroidism, and those with a history of diabetes or who were taking hypoglycemic agents were excluded from the study. After 17 participants were excluded, a total of 919 participants with complete data were enrolled in the study.

### 2.2. Sample Collection

A questionnaire was given to each participant to obtain general information as well as information on history of thyroid disease, diabetes, hypertension, and dyslipidemia with the associated treatments. Anthropometric data were collected by qualified physicians. Height and weight were measured to calculate body mass index (BMI): BMI = weight (kg)/height² (m²). With the subjects standing with their feet 25–30 cm apart, waist circumference was measured by placing the measuring tape horizontally around the patient's abdomen from the horizontal waistline of the two middle points of the anterosuperior iliac spine through the inferior margin of 12th ribs with the tape held tightly to the skin, but without pressing. The measurement precision was ±0.1 cm. The thyroid gland was examined by qualified clinical endocrinologists according to the standard procedure combined with inspection and palpation. Systolic blood pressure (SBP) and diastolic blood pressure (DBP) were measured twice after a 10-minute rest with the patient in the sitting position using a mercury-gravity sphygmomanometer. There was a 2-minute interval between the two measurements, and the mean value of the two measurements was used. Blood samples were obtained after an overnight fast lasting at least 10 hours; a portion of the samples were sent to the laboratory in the Liaoyang Diabetes Hospital within 30 minutes of collection for measurement of fasting plasma glucose (FPG), total cholesterol (TC), low density lipoprotein cholesterol (LDL-C), high density lipoprotein cholesterol (HDL-C), and triglycerides (TG). The serum samples for fasting insulin (FINS), TSH, and thyroid peroxidase antibodies (TPOAb) were stored at −80°C until analysis. The Homoeostasis Model of Insulin Resistance (HOMA-IR) score was calculated using the following formula: HOMA-IR = (FPG [mmol/L] × insulin [*µ*U/mL])/22.5.

### 2.3. Biochemical Examinations

Serum TSH and TPOAb were tested using an electrochemiluminescence immunoassay (Roche Cobas 6000, Roche Ltd, Switzerland). FPG was measured using the glucose oxidase method. TC, LDL-C, HDL-C, and TG were measured by routine enzymatic methods (Olympus 400, Olympus Optical Company, Tokyo, Japan). Insulin was determined by radioimmunoassay (China Institute of Atomic Energy, Beijing, China).

### 2.4. Diagnostic and Grouping Criteria

Subclinical thyroid diseases: the TSH reference range (1.01–5.73 mIU/L) was set by the National Academy of Clinical Biochemistry (NACB) guidelines [9]. According to the manufacturer's manual (Diagnostic Products of Roche), the reference range for subclinical hypothyroidism was defined as TSH > 5.73 mIU/L and FT4 levels within the reference range. Subclinical hyperthyroidism was defined as TSH < 1.01 mIU/L and FT3 and FT4 levels within the reference range. Based on the results of thyroid function testing, the participants were classified as subclinical hyperthyroidism, euthyroidism, or subclinical hypothyroidism. Participants who considered euthyroid were further divided into low-normal (TSH 1.01–2.14 mIU/L), mid-range (TSH 2.15–3.07 mIU/L), and high-normal (TSH 3.08–5.72  mIU/L) groups.

*Metabolic Syndrome*. The International Diabetes Federation (IDF) definition of metabolic syndrome in children and adolescents [10] was used in this study. The following criteria were used for children: (1) age 10 to 16 years, obesity ≥ 90th percentile as assessed by waist circumference [11] and at least two of the following: TG ≥ 1.70 mmol/L, HDL-C < 1.03 mmol/L, SBP ≥ 130 mmHg, or DBP ≥ 85 mmHg, and FPG ≥ 5.6 mmol/L or known type 2 diabetes mellitus; (2) for age ≥ 16 years, criteria included obesity ≥ 90th percentile as assessed by waist circumference and at least two of the following: TG ≥ 1.70 mmol/L or receiving therapy, HDL-C < 1.03 mmol/L (male) or <1.29 mmol/L (female) or receiving therapy, SBP ≥ 130 mmHg or DBP ≥ 85 mmHg or receiving therapy, and FPG ≥ 5.6 mmol/L or known type 2 diabetes mellitus. Using the IDF diagnostic criteria for metabolic syndrome, the participants were divided into two groups: metabolic syndrome or nonmetabolic syndrome.

### 2.5. Statistical Analysis

Data processing and statistical analysis were performed using SPSS 17.0 software. Data are expressed as mean ± standard deviation (SD) or median (interquartile range). Statistical comparisons were performed using independent-samples* t*-tests, Kruskal-Wallis test for percentages, and analysis of variance (ANOVA) when more than two groups were compared. Covariance analysis was used for further adjustments. Multiple linear regression was used to evaluate the correlations among factors. TSH, FPG, TG, FINS, and HOMA-IR scores were analyzed after transformation. Logistic regression was used to analyze multiplicity. *P* < 0.05 was considered statistically significant.

## 3. Results

### 3.1. Comparison of Metabolic Syndrome Components in Groups with Different Serum TSH Levels

Waist circumference (76.4 ± 10.7 versus 70.0 ± 10.6 cm, *P* = 0.006) and BMI (23.90 ± 4.20 versus 21.51 ± 4.16 kg/m^2^, *P* = 0.011) were significantly higher in the subclinical hypothyroid group than in the euthyroid group. The level of TG (0.95 (0.68–1.30) versus 0.63 (0.52–1.16) mmol/L, *P* = 0.002) was significantly higher in the euthyroid group than in the subclinical hyperthyroid group (Table 1). The prevalence of obesity differed significantly among the three groups (*P* = 0.002), with a risk of obesity in the subclinical hypothyroid group being 3.444 times higher than that of the euthyroid group (odds ratio (OR) = 3.444, 95% confidence interval (CI): 1.570–7.553; Table 2). In the multiple linear regression model, there was a significant positive correlation between TSH and waist circumference (*β* = 1.512, *P* = 0.019) and between TSH and BMI (*β* = 0.562, *P* = 0.024), TC (*β* = 0.160, *P* = 0.003), LDL-C (*β* = 0.032, *P* = 0.008), or TG (*β* = 0.095, *P* = 0.001). However, the strength of these associations was less than that of the correlation between TSH and waist circumference (Table 6).

### 3.2. Comparison of TSH Levels between Metabolic Syndrome and Nonmetabolic Syndrome Groups

Based on the IDF criteria for diagnosis, the prevalence of metabolic syndrome in adolescents was 8.0% (72.2% were male). After adjusting for age and gender, the TSH level in the metabolic syndrome group was significantly higher than that in nonmetabolic syndrome group (2.65 (2.28–3.80) versus 2.53 (1.92–3.45) mIU/L, *P* = 0.032). TSH levels were also higher among participants with waist circumference ≥90th percentile than those with normal waist circumference (2.57 (2.01–3.70) versus 2.55 (1.91–3.41) mIU/L, *P* = 0.026) and were higher among participants with hypertriglyceridemia than those with normal TG levels (2.76 (2.13–3.77) versus 2.50 (1.90–3.42) mIU/L, *P* = 0.037). When participants were grouped by blood pressure (hypertensive or not), HDL-C levels, and FPG (hyperglycemic or not), TSH levels were not significantly different (*P* > 0.05; Table 3).

### 3.3. Comparison of the Components of Metabolic Syndrome in Groups with Normal TSH Levels

The levels of TG in the high-normal TSH group were significantly higher than those in the low-normal TSH group (1.02 (0.71–1.42) versus 0.88 (0.63–1.25) mmol/L, *P* = 0.006; Table 4). The risk of having low HDL-C levels in the low-normal TSH group was lower than that in the mid-range TSH group (OR = 0.681, 95% CI: 0.480–0.967; Table 5). In the multiple linear regression model, a significant positive correlation was found between normal range TSH and DBP (*β* = 1.991, *P* = 0.048); however, after adjusting for age, gender, HOMA-IR scores, and BMI, the correlation was no longer present (*β* = 1.692, *P* = 0.093). There was also a positive correlation between normal range TSH and TC (*β* = 0.173, *P* = 0.013), LDL-C (*β* = 0.031, *P* = 0.043), or TG (*β* = 0.132, *P* = 0.021). We did not find any correlation between the prevalence of metabolic syndrome and its components and normal TSH. However, no correlation was found between normal range TSH and waist circumference, BMI, SBP, FPG, HDL-C, FINS, or HOMA-IR scores (*P* > 0.05; Table 6).

## 4. Discussion

Our study showed that among 919 participants, 866 (94.2%) had normal thyroid function, 29 (3.2%) had subclinical hypothyroidism, and 24 (2.6%) had subclinical hyperthyroidism. We found that the TSH level in the metabolic syndrome group was significantly higher than that in the non-metabolic syndrome group (Table 3). Our study indicated that the increase in serum TSH was positively correlated with the metabolic syndrome components of obesity and dyslipidemia in adolescents in Liaoyang City. However, whether these conditions can be recognized as risk factors for metabolic syndrome warrants further study.

Among the 919 adolescents, those with a waist circumference at the 90th percentile or above were regarded as obese; the total percentage meeting this criterion was 23.0%, which further confirmed the high prevalence of obesity in adolescents. We speculate that leptin may play an important role in the development of physiological insulin resistance, which is a major component of metabolic syndrome. Soliman et al. [12] reported that leptin resistance is associated with insulin resistance and abdominal obesity. Significant changes in leptin levels are observed during the progressive pubertal stages, with a distinct dimorphism between boys and girls [12]. In our study, there was a positive correlation between increased TSH and obesity. We found that waist circumference and BMI were significantly higher in the subclinical hypothyroid group than in the euthyroid group, and they both were increased with an increase in TSH. Moreover, the risk of obesity in participants with subclinical hypothyroidism was 3.444 times higher than that in the euthyroid group. Knudsen et al. [13] found a positive correlation between BMI and TSH in adults. Bastemir et al. [14] also revealed a positive relationship between TSH and body weight, BMI, and waist circumference, and serum TSH levels were higher in the obese than in lean subjects. In our study, we found that TSH levels were higher among participants with waist circumference ≥90th percentile than those with normal waist circumference. Meanwhile, TSH within the normal range was not correlated with either waist circumference or BMI. Our data suggest that the positive correlation between TSH and obesity may become more obvious with higher TSH levels exceeding the normal range. However, the cause-effect relationship between thyroid dysfunction and obesity remains controversial. Thyroid hormones regulate both energy consumption and basal metabolic rates; in addition, low basal metabolic rate is a risk factor for obesity. The question remains as to whether the changes in the levels of thyroid hormone and TSH in obesity are the cause or result of weight status. Bianco and McAninch [15] reported that thyroid-hormone signaling, particularly through induction of type II deiodinase, plays a central role in brown adipogenesis* in vitro* and in brown adipose tissue development in mouse embryos. Recent studies have found adipocytes and preadipocytes express TSH receptors. Studies* in vitro* and* in vivo* have demonstrated that the action of TSH via its receptors in fat tissue induces differentiation of preadipocytes into adipocytes and expansion of adipose tissue (adipogenesis) [14]. However, administration of thyroxine to obese children with moderately elevated TSH levels does not alter either weight status or lipid profiles [16]. The abnormalities in thyroid function and TSH are mostly normalized after weight loss [17–19]. As a result, the TSH increase in obese patients seems to be a consequence rather than a cause of obesity. There are several postulated causes of increased TSH levels in obesity, including autoimmune status, leptin levels, and inflammatory factors. The most favored hypothesis attributes the elevated TSH levels in obesity to increased leptin-mediated production of pro-thyroid releasing hormone [20]. We will evaluate leptin levels, inflammatory factors, and autoimmune antibodies in obese adolescents in future studies.

In our current study, a correlation also was found between elevated TSH and dyslipidemia. With every 1 mIU/L increase in TSH, TG was increased by 0.095 mmol/L; this positive linear correlation also existed between normal range TSH and TG. Among patients with hypertriglyceridemia, TSH levels were higher than in participants with normal TG levels. The above findings illustrate the positive relationship between TSH and TG. Regardless of whether TSH was within the normal range, there were positive linear correlations between TSH and TC as well as TSH and LDL-C, which indicated that both TC and LDL-C could be increased with an increase in TSH. For HDL-C, the risk of low HDL-C in the low-normal TSH group was less than that in the mid-range TSH group (OR = 0.681, 95% CI: 0.480–0.967), which indicated that a decrease in TSH within the normal range could reduce the risk of low levels of HDL-C.

Thyroid hormones influence various metabolic pathways, and both the composition and transport of lipoproteins are impaired in thyroid diseases. Hypothyroidism is a major cause of secondary dyslipidemia, which is characterized by hypercholesterolemia and a marked increase in LDL-C, which results from decreased fractional clearance by the reduced number of LDL receptors in the liver. Althaus et al. [21] found that in subjects with subclinical hypothyroidism, serum concentrations of LDL-C are significantly higher and HDL-C levels are lower compared with controls. However, another study showed no difference in the levels of TC, TG, and HDL-C between subjects with subclinical hypothyroidism and euthyroidism [22]. Although different studies have provided conflicting results, our data confirmed that elevated TSH, whether in the normal range or not, can cause dyslipidemia in adolescents.

We also found the TSH level in the metabolic syndrome group was significantly higher than that in non-metabolic syndrome group, in agreement with the results of our previous study, which showed that serum TSH is higher among adult individuals with metabolic syndrome than among those without metabolic syndrome [23]. Ayturk et al. [24] also reported a significant positive correlation between TSH and metabolic syndrome in adults. In general, most studies attribute metabolic syndrome to insulin resistance, and central obesity is one cause of insulin resistance. Our study indicated the increased serum TSH in adolescents was associated with obesity and dyslipidemia. It is possible that TSH differentially influences the different components of metabolic syndrome. Further studies should be carried out to determine whether TSH should be measured in adolescents with metabolic syndrome.

We also found a significant positive correlation between normal range TSH and DBP, with an increase in DBP of 1.991 mmHg for every 1 mIU/L increase in TSH. However, after adjusting for age, gender, HOMA-IR scores, and BMI, the correlation disappeared. Moreover, this relationship was not seen for SBP. Meanwhile, no difference was found for SBP, DBP, or the risk of hypertension among groups with different levels of TSH. The relationship between TSH and blood pressure is not clear yet. Åsvold et al. [25] demonstrated positive and linear correlations between normal range TSH and SBP as well as normal range TSH and DBP, whereas other researchers could not confirm these findings [8, 22]. The underlying mechanisms for the relationship between TSH and blood pressure are not fully understood, and further studies are needed.

Our study did not show a correlation between TSH and FPG, possibly because adolescents with overt hyperthyroidism or hypothyroidism were excluded. All the participants had normal FT_3_ and FT_4_, and the effects of thyroxine were not strong enough to influence FPG. Additionally, we used a cross-sectional design in our study, which did not address the question of whether adolescents with subclinical hypothyroidism should receive thyroid hormone replacement therapy. In summary, we found that an increase in serum TSH was positively correlated with components of metabolic syndrome and might be a risk factor for metabolic syndrome in adolescents. Further investigations are essential to further confirm the relationship between TSH and components of metabolic syndrome in adolescents as well as the underlying mechanism(s).

## Figures and Tables

**Table 1:** Comparison of metabolic syndrome and its components in groups with different serum TSH levels.

	TSH (mIU/L)			*P*
	<1.01 (*n* = 24)	1.01–5.73 (*n* = 866)	>5.73 (*n* = 29)	
Age (yr)	14 ± 2	14 ± 1	14 ± 2	0.689
Waist circumference (cm)	71.3 ± 10.9	70.0 ± 10.6	76.4 ± 10.7∗	0.021
BMI (kg/m^2^)	21.68 ± 4.58	21.51 ± 4.16	23.90 ± 4.20∗	0.040
FPG (mmol/L)	4.70 (4.50–5.10)	4.80 (4.40–5.00)	4.60 (4.40–5.10)	0.803
TC (mmol/L)	4.19 ± 0.85	4.49 ± 0.80	4.62 ± 0.90	0.160
HDL-C (mmol/L)	1.01 ± 0.25	1.06 ± 0.26	1.04 ± 0.21	0.666
LDL-C (mmol/L)	3.30 ± 0.13	3.37 ± 0.17	3.40 ± 0.27	0.136
TG (mmol/L)	0.63 (0.52–1.16)∗	0.95 (0.68–1.30)	0.82 (0.66–1.37)	0.006
SBP (mmHg)	117 ± 14	114 ± 14	113 ± 12	0.285
DBP (mmHg)	69 ± 12	70 ± 11	69 ± 11	0.508
FINS (*µ*IU/mL)	19.00 (15.20–24.50)	18.00 (13.00–24.00)	21.00 (18.75–26.50)	0.803
HOMA-IR	3.88 (3.09–5.47)	3.76 (2.72–5.21)	4.20 (3.87–5.48)	0.939

Data are presented as mean ± SD or median (interquartile range); compared with euthyroid group, *P < 0.05; age, after adjusting for gender; waist circumference and BMI, after adjusting for age, gender, and HOMA-IR; HOMA-IR, after adjusting for age, gender, and BMI; others, after adjusting for age, gender, HOMA-IR, and BMI.

**Table 2:** Comparison of the prevalence of metabolic syndrome and its components in groups with different serum TSH levels.

	TSH (mIU/L)			*P*
	<1.01 (*n* = 24)	1.01–5.73 (*n* = 866)	>5.73 (*n* = 29)	
Overweight/obesity				
Prevalence (%)	30.4	22.0	50.0	0.002
OR	1.470	Ref.	3.444∗	
Hyperglycemia				
Prevalence (%)	4.2	6.4	10.3	0.621
OR	0.615	Ref.	2.636	
Hypertension				
Prevalence (%)	16.7	17.6	10.7	0.633
OR	0.853	Ref.	0.362	
Low HDL-C				
Prevalence (%)	54.2	46.3	53.6	0.574
OR	1.346	Ref.	1.052	
High TG				
Prevalence (%)	8.3	9.8	13.8	0.754
OR	0.831	Ref.	1.246	
Metabolic syndrome				
Prevalence (%)	8.7	7.7	14.3	0.449
OR	1.117	Ref.	1.128	

Ref. is control group; compared with euthyroid group, *P < 0.05; the prevalence of overweight/obesity, after adjusting for age, gender, and HOMA-IR; others, after adjusting for age, gender, HOMA-IR, and BMI.

**Table 3:** Comparison of serum TSH levels in different components of metabolic syndrome between the case and control groups.

	Overweight/obesity	Hyperglycemia	Hypertension	Low HDL-C	High TG	Metabolic syndrome
TSH (mIU/L)						
Case group	2.57 (2.01–3.70)	2.62 (2.01–3.45)	2.61 (1.97–3.55)	2.58 (2.00–3.60)	2.76 (2.13–3.77)	2.65 (2.28–3.80)
Control group	2.55 (1.91–3.41)	2.55 (1.92–3.46)	2.50 (1.92–3.45)	2.50 (1.85–3.34)	2.50 (1.90–3.42)	2.53 (1.92–3.45)

*P*	0.026∗	0.481	0.314	0.151	0.037∗	0.032∗

Data are presented as median (interquartile range); *P < 0.05; MS and its components, after adjusting for age and gender.

**Table 4:** Comparison of the components of metabolic syndrome in groups with normal TSH levels.

	TSH (mIU/L)			*P*
	Low-normal TSH group 1.01–2.14 (n = 289)	Mid-range TSH group 2.15–3.07 (n = 287)	High-normal TSH group 3.08–5.72 (n = 290)	
Age (yr)	14 ± 1	14 ± 1	14 ± 1	0.338
Waist circumference (cm)	69.3 ± 10.4	70.5 ± 10.4	70.3 ± 10.9	0.287
BMI (kg/m^2^)	21.25 ± 3.88	21.60 ± 4.02	21.67 ± 4.55	0.381
FPG (mmol/L)	4.70 (4.40–5.01)	4.80 (4.40–5.00)	4.80 (4.40–5.00)	0.849
TC (mmol/L)	4.41 ± 0.73	4.50 ± 0.82	4.55 ± 0.83	0.177
HDL-C (mmol/L)	1.08 ± 0.27	1.06 ± 0.26	1.05 ± 0.26	0.377
LDL-C (mmol/L)	3.35 ± 0.15	3.37 ± 0.17	3.38 ± 0.19	0.075
TG (mmol/L)	0.88 (0.63–1.25)	0.95 (0.68–1.27)	1.02 (0.71–1.42)∗	0.022
SBP (mmHg)	114 ± 13	113 ± 15	116 ± 14	0.051
DBP (mmHg)	70 ± 12	69 ± 12	71 ± 10	0.079
FINS (*µ*IU/mL)	18.00 (13.43–24.00)	18.00 (13.00–25.00)	18.00 (13.00–26.00)	0.849
HOMA-IR	3.75 (2.70–4.92)	3.76 (2.73–5.22)	3.77 (2.69–5.43)	0.986

Data are presented as mean ± SD or median (interquartile range); compared with low-normal TSH group, *P < 0.05; age, after adjusting for gender; waist circumference, BMI, after adjusting for age, gender, and HOMA-IR; HOMA-IR, after adjusting for age, gender, and BMI; others, after adjusting for age, gender, HOMA-IR, and BMI.

**Table 5:** Comparison of the prevalence of metabolic syndrome and its components in groups with normal TSH levels.

	TSH (mIU/L)			*P*
	1.01–2.14 (n = 289)	2.15–3.07 (n = 287)	3.08–5.72 (n = 290)	
Overweight/obesity				
Prevalence (%)	19.4	24.6	21.9	0.315
OR	0.752	Ref.	0.827	
Hyperglycemia				
Prevalence (%)	5.9	6.6	6.6	0.923
OR	0.988	Ref.	0.990	
Hypertension				
Prevalence (%)	15.7	17.4	19.8	0.439
OR	0.931	Ref.	1.166	
Low HDL-C				
Prevalence (%)	40.8	48.9	49.3	0.070
OR	0.681∗	Ref.	1.020	
High TG				
Prevalence (%)	7.3	10.5	11.7	0.179
OR	0.728	Ref.	1.004	
MS				
Prevalence (%)	4.9	9.2	9.0	0.099
OR	0.618	Ref.	0.722	

Ref. is control group; compared with mid-range TSH group, *P < 0.05; the prevalence of overweight/obesity, after adjusting for age, gender, and HOMA-IR; others, after adjusting for age, gender, HOMA-IR, and BMI.

**Table 6:** The relationship between TSH and the components of metabolic syndrome by multiple linear regression model.

	Model	*β*	*P*
Waist circumference			
T	1	1.512	0.019∗
N	1	1.206	0.152
BMI			
T	1	0.562	0.024∗
N	1	0.383	0.240
TC			
T	2	0.160	0.003∗
N	2	0.173	0.013∗
LDL-C			
T	2	0.032	0.008∗
N	2	0.031	0.043∗
HDL-C			
T	2	−0.002	0.923
N	2	−0.020	0.379
TG			
T	2	0.095	0.001∗
N	2	0.132	0.021∗
FPG			
T	2	0.002	0.766
N	2	0.005	0.613
SBP			
T	2	−0.139	0.875
N	2	1.216	0.296
DBP			
T	2	0.675	0.377
N	2	1.692	0.093
FINS			
T	2	−0.002	0.766
N	2	−0.005	0.613
HOMA-IR			
T	3	0.010	0.745
N	3	0.017	0.679

*P < 0.05; values of *β* are regression coefficients; T: total TSH; N: TSH within normal range; model 1: adjusted for age, gender, and HOMA-IR; model 2: adjusted for age, gender, HOMA-IR, and BMI; model 3: adjusted for age, gender, and BMI.

## References

[1] Gu D, Reynolds K, Wu X (2005). Prevalence of the metabolic syndrome and overweight among adults in China. *The Lancet*.

[2] Wan NJ, Mi J, Wang TY (2007). Metabolic syndrome in overweight and obese schoolchildren in Beijing. *Chinese Journal of Pediatrics*.

[3] Hoogwerf BJ, Nutall FQ (1984). Long-term weight regulation in treated hyperthyroid and hypothyroid subjects. *The American Journal of Medicine*.

[4] Liu D, Jiang F, Shan Z (2010). A cross-sectional survey of relationship between serum TSH level and blood pressure. *Journal of Human Hypertension*.

[5] Duntas LH (2002). Thyroid disease and lipids. *Thyroid*.

[6] de Pergola G, Giorgino F, Benigno R, Guida P, Giorgino R (2008). Independent influence of insulin, catecholamines, and thyroid hormones on metabolic syndrome. *Obesity*.

[7] Taneichi H, Sasai T, Ohara M (2011). Higher serum free triiodothyronine levels within the normal range are associated with metabolic syndrome components in type 2 diabetic subjects with euthyroidism. *Tohoku Journal of Experimental Medicine*.

[8] Roos A, Bakker SJL, Links TP, Gans ROB, Wolffenbuttel BHR (2007). Thyroid function is associated with components of the metabolic syndrome in euthyroid subjects. *Journal of Clinical Endocrinology and Metabolism*.

[9] Baloch Z, Carayon P, Conte-Devolx B (2003). Laboratory medicine practice guidelines. Laboratory support for the diagnosis and monitoring of thyroid disease.. *Thyroid*.

[10] Zimmet P, Alberti G, Kaufman F (2007). The metabolic syndrome in children and adolescents. *The Lancet*.

[11] Ma G, Ji C, Ma J (2010). Waist circumference reference values for screening cardiovascular risk factors in Chinese children and adolescents aged 7—18 years. *Zhonghua liuxingbingxue zazhi*.

[12] Soliman AT, Yasin M, Kassem A (2012). Leptin in pediatrics: a hormone from adipocyte that wheels several functions in children. *Indian Journal of Endocrinology and Metabolism*.

[13] Knudsen N, Laurberg P, Rasmussen LB (2005). Small differences in thyroid function may be important for body mass index and the occurrence of obesity in the population. *The Journal of Clinical Endocrinology and Metabolism*.

[14] Bastemir M, Akin F, Alkis E, Kaptanoqlu BB (2007). Obesity is associated with increased serum TSH level, independent of thyroid function. *Swiss Medical Weekly*.

[15] Bianco AC, McAninch EA (2013). The role of thyroid hormone and brown adipose tissue in energy homoeostasis. *The Lancet Diabetes and Endocrinology*.

[16] Krude H, Biebermann H, Schnabel D (2003). Obesity due to proopiomelanocortin deficiency: three new cases and treatment trials with thyroid hormone and ACTH4-10. *The Journal of Clinical Endocrinology & Metabolism*.

[17] Reinehr T, Andler W (2002). Thyroid hormones before and after weight loss in obesity. *Archives of Disease in Childhood*.

[18] Shalitin S, Yackobovitch-Gavan M, Phillip M (2009). Prevalence of thyroid dysfunction in obese children and adolescents before and after weight reduction and its relation to other metabolic parameters. *Hormone Research*.

[19] Reinehr T, Isa A, de Sousa G, Dieffenbach R, Andler W (2008). Thyroid hormones and their relation to weight status. *Hormone Research*.

[20] Reinehr T (2011). Thyroid function in the nutritionally obese child and adolescent. *Current Opinion in Pediatrics*.

[21] Althaus BU, Staub J-J, Ryff-de Leche A, Oberhansli A, Stahelin HB (1988). LDL/HDL-changes in subclinical hypothyroidism: possible risk factors for coronary heart disease. *Clinical Endocrinology*.

[22] Takashima N, Niwa Y, Mannami T, Tomoike H, Iwai N (2007). Characterization of subclinical thyroid dysfunction from cardiovascular and metabolic viewpoints. *Circulation Journal*.

[23] Lai Y, Wang J, Jiang F (2011). The relationship between serum thyrotropin and components of metabolic syndrome. *Endocrine Journal*.

[24] Ayturk S, Gursoy A, Kut A, Anil C, Nar A, Tutuncu NB (2009). Metabolic syndrome and its components are associated with increased thyroid volume and nodule prevalence in a mild-to-moderate iodine-deficient area. *European Journal of Endocrinology*.

[25] Åsvold BO, Bjøro T, Nilsen TI, Vatten LJ (2007). Association between blood pressure and serum thyroid-stimulating hormone concentration within the reference range: a population-based study. *The Journal of Clinical Endocrinology & Metabolism*.

